# 3-[(*N*-Methyl­anilino)meth­yl]-5-(thio­phen-2-yl)-1,3,4-oxadiazole-2(3*H*)-thione

**DOI:** 10.1107/S1600536812014419

**Published:** 2012-04-13

**Authors:** Ali A. El-Emam, Mohamed A. Al-Omar, Hazem A. Ghabbour, Hoong-Kun Fun, Tze Shyang Chia

**Affiliations:** a Department of Pharmaceutical Chemistry, College of Pharmacy, King Saud University, Riyadh 11451, Saudi Arabia; b X-ray Crystallography Unit, School of Physics, Universiti Sains Malaysia, 11800 USM, Penang, Malaysia

## Abstract

In the title compound, C_14_H_13_N_3_OS_2_, the thio­phene ring is disordered over two orientations by *ca* 180° about the C—C bond axis linking the ring to the rest of the mol­ecule, with a site-occupancy ratio of 0.651 (5):0.349 (5). The central 1,3,4-oxadiazole-2(3*H*)-thione ring forms dihedral angles of 9.2 (5), 4.6 (11) and 47.70 (7)° with the major and minor parts of the disordered thio­phene ring and the terminal phenyl ring, respectively. In the crystal, no significant inter­molecular hydrogen bonds are observed. The crystal packing is stabilized by π–π inter­actions [centroid–centroid distance = 3.589 (2) Å].

## Related literature

For the biological activity of 1,3,4-oxadiazole derivatives, see: Navarrete-Vázquez *et al.* (2007 ▶); Kadi *et al.* (2007 ▶); Padmavathi *et al.* (2009 ▶); El-Emam *et al.* (2004 ▶); Al-Deeb *et al.* (2006 ▶). For the synthesis of the title compound, see: Al-Omar (2010 ▶). For related 1,3,4-oxadiazole structures, see: Fun *et al.* (2011 ▶); El-Emam *et al.* (2012 ▶).
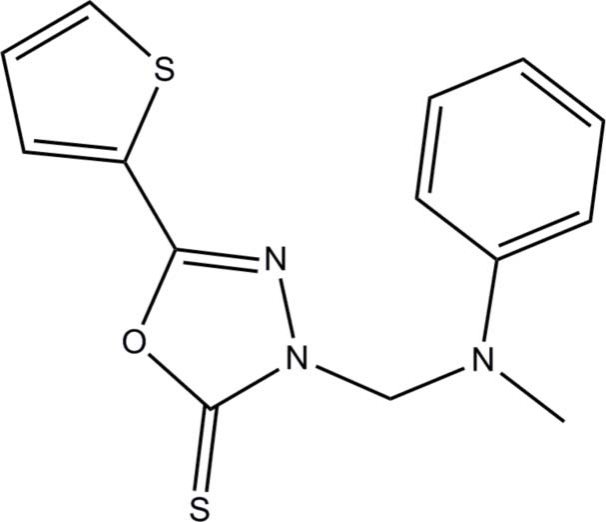


## Experimental

### 

#### Crystal data

C_14_H_13_N_3_OS_2_
*M* *_r_* = 303.39
Monoclinic, 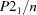

*a* = 11.9682 (8) Å
*b* = 7.4526 (5) Å
*c* = 17.0749 (14) Å
β = 108.072 (6)°
*V* = 1447.85 (18) Å^3^
*Z* = 4
Cu *K*α radiation
μ = 3.32 mm^−1^
*T* = 296 K
0.92 × 0.16 × 0.09 mm

#### Data collection

Bruker SMART APEXII CCD area-detector diffractometer
Absorption correction: multi-scan (*SADABS*; Bruker, 2009 ▶) *T*_min_ = 0.150, *T*_max_ = 0.754
10021 measured reflections
2676 independent reflections
1516 reflections with *I* > 2σ(*I*)
*R*_int_ = 0.066

#### Refinement

*R*[*F* ^2^ > 2σ(*F* ^2^)] = 0.048
*wR*(*F* ^2^) = 0.156
*S* = 0.97
2676 reflections
220 parameters
H-atom parameters constrained
Δρ_max_ = 0.16 e Å^−3^
Δρ_min_ = −0.20 e Å^−3^

### 

Data collection: *APEX2* (Bruker, 2009 ▶); cell refinement: *SAINT* (Bruker, 2009 ▶); data reduction: *SAINT*; program(s) used to solve structure: *SHELXTL* (Sheldrick, 2008 ▶); program(s) used to refine structure: *SHELXTL*; molecular graphics: *SHELXTL*; software used to prepare material for publication: *SHELXTL* and *PLATON* (Spek, 2009 ▶).

## Supplementary Material

Crystal structure: contains datablock(s) global, I. DOI: 10.1107/S1600536812014419/is5107sup1.cif

Structure factors: contains datablock(s) I. DOI: 10.1107/S1600536812014419/is5107Isup2.hkl

Supplementary material file. DOI: 10.1107/S1600536812014419/is5107Isup3.cml

Additional supplementary materials:  crystallographic information; 3D view; checkCIF report

## Figures and Tables

**Fig. 1.**
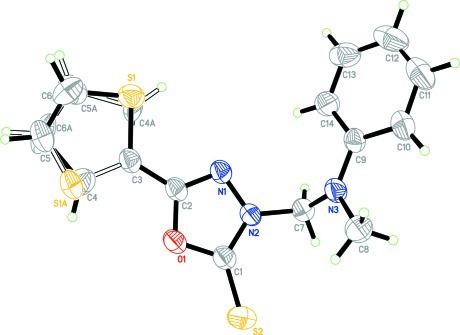
The molecular structure of the title compound with atom labels and 30% probability displacement ellipsoids. Atoms of the minor occupancy component are labelled with the suffix A.

## supplementary crystallographic information

### Comment

Considerable attention has been devoted to 1,3,4-oxadiazole derivatives which have long been known for their diverse chemotherapeutic properties as antiviral agents against the HIV-1 viruses (El-Emam *et al.*, 2004), antibacterial agents (Navarrete-Vázquez *et al.*, 2007; Padmavathi *et al.*, 2009) and anti-inflammatory agents (Kadi *et al.*, 2007; Al-Deeb *et al.*, 2006). The title compound (I) was synthesized among a series of 2-thienyl-1,3,4-oxadiazoles and related derivatives as potential antimicrobial agents (Al-Omar, 2010).

The molecular structure of the title compound is shown in Fig. 1. The thiophene ring is disordered by *ca.* 180° rotation about the C2—C3 bond axis with a site-occupancy ratio of 0.651 (5):0.349 (5). The central 1,3,4-oxadiazole-2(3*H*)-thione ring (N1/N2/C1/O1/C2/S2; maximum deviation = 0.0157 (12) Å at atom S2) forms dihedral angles of 9.23 (51), 4.6 (11) and 47.70 (7)° with the major and minor parts of the disordered thiophene ring [S1/C3–C6: maximum deviation = 0.024 (11) Å at atom C4 and S1A/C3/C4A–C6A: maximum deviation = 0.04 (3) Å at atom C6A] and the terminal phenyl ring (C9–C14), respectively.

In the crystal packing, no significant intermolecular hydrogen bondings are observed. The crystal packing is stabilized by a π–π interaction with *Cg*2···*Cg*4 distance = 3.589 (2) Å (symmetry code: 3/2-*x*, -1/2+*y*, 3/2-*z*), where *Cg*2 and *Cg*4 are the centroids of O1/C1/C2/N1/N2 and C9–C14 rings, respectively.

### Experimental

*N*-Methylaniline (214 mg, 2 mmol) and 37% formaldehyde solution (0.5 ml) were added to a solution of 5-(thiophen-2-yl)-1,3,4-oxadiazole-2-thiol (369 mg, 2 mmol) in ethanol (8 ml). The mixture was stirred at room temperature for 2 h and allowed to stand overnight. The precipitated crude product was filtered, washed with cold ethanol, dried, and crystallized from ethanol to yield 558 mg (92%) of the title compound (I) as colorless needle crystals. *M*.p.: 112–114 °C. ^1^H NMR (CDCl_3_, 500.13 MHz): δ 3.28 (s, 3H, CH_3_), 5.64 (d, 2H, NCH_2_N), 6.83–7.36 (m, 6H, Ar—H & Thiophene-H), 7.55 (d, 1H, Thiophene-H, *J* = 5.0 Hz), 7.70 (d, 1H, Thiophene-H, *J* = 5.0 Hz). ^13^C NMR (CDCl_3_, 125.76 MHz): δ 39.54 (CH_3_), 66.80 (CH_2_), 113.72, 119.12, 123.65, 128.30, 129.36, 130.36, 131.07, 146.69 (Ar—C & Thiophene-C), 155.89 (Oxadiazole C-5), 176.32 (C=S).

### Refinement

All H atoms were positioned geometrically (C—H = 0.93, 0.96 or 0.97 Å) and refined using a riding model with *U*_iso_(H) = 1.2 or 1.5*U*_eq_(C). A rotating group model was applied to the methyl group. Initially similarity and FLAT (only to the minor component) restraints were used. In the final refinement, these restraints were removed and the ratio of the refined site occupancies for the major and minor components of the disordered thiophene ring is 0.651 (5):0.349 (5).

### Crystal data

**Table:**

C_14_H_13_N_3_OS_2_	*F*(000) = 632
*M**_r_* = 303.39	*D*_x_ = 1.392 Mg m^−^^3^
Monoclinic, *P*2_1_/*n*	Cu *K*α radiation, λ = 1.54178 Å
Hall symbol: -P 2yn	Cell parameters from 587 reflections
*a* = 11.9682 (8) Å	θ = 4.0–45.3°
*b* = 7.4526 (5) Å	µ = 3.32 mm^−^^1^
*c* = 17.0749 (14) Å	*T* = 296 K
β = 108.072 (6)°	Needle, colorless
*V* = 1447.85 (18) Å^3^	0.92 × 0.16 × 0.09 mm
*Z* = 4	

### Data collection

**Table:**

Bruker SMART APEXII CCD area-detector diffractometer	2676 independent reflections
Radiation source: fine-focus sealed tube	1516 reflections with *I* > 2σ(*I*)
Graphite monochromator	*R*_int_ = 0.066
φ and ω scans	θ_max_ = 69.5°, θ_min_ = 4.0°
Absorption correction: multi-scan (*SADABS*; Bruker, 2009)	*h* = −14→14
*T*_min_ = 0.150, *T*_max_ = 0.754	*k* = −8→7
10021 measured reflections	*l* = −20→18

### Refinement

**Table:**

Refinement on *F*^2^	Secondary atom site location: difference Fourier map
Least-squares matrix: full	Hydrogen site location: inferred from neighbouring sites
*R*[*F*^2^ > 2σ(*F*^2^)] = 0.048	H-atom parameters constrained
*wR*(*F*^2^) = 0.156	*w* = 1/[σ^2^(*F*_o_^2^) + (0.0811*P*)^2^] where *P* = (*F*_o_^2^ + 2*F*_c_^2^)/3
*S* = 0.97	(Δ/σ)_max_ = 0.001
2676 reflections	Δρ_max_ = 0.16 e Å^−^^3^
220 parameters	Δρ_min_ = −0.20 e Å^−^^3^
0 restraints	Extinction correction: *SHELXTL* (Sheldrick, 2008), Fc^*^=kFc[1+0.001xFc^2^λ^3^/sin(2θ)]^-1/4^
Primary atom site location: structure-invariant direct methods	Extinction coefficient: 0.0019 (4)

### Special details

**Table:**

Geometry. All e.s.d.'s (except the e.s.d. in the dihedral angle between two l.s. planes) are estimated using the full covariance matrix. The cell e.s.d.'s are taken into account individually in the estimation of e.s.d.'s in distances, angles and torsion angles; correlations between e.s.d.'s in cell parameters are only used when they are defined by crystal symmetry. An approximate (isotropic) treatment of cell e.s.d.'s is used for estimating e.s.d.'s involving l.s. planes.
Refinement. Refinement of *F*^2^ against ALL reflections. The weighted *R*-factor *wR* and goodness of fit *S* are based on *F*^2^, conventional *R*-factors *R* are based on *F*, with *F* set to zero for negative *F*^2^. The threshold expression of *F*^2^ > σ(*F*^2^) is used only for calculating *R*-factors(gt) *etc*. and is not relevant to the choice of reflections for refinement. *R*-factors based on *F*^2^ are statistically about twice as large as those based on *F*, and *R*- factors based on ALL data will be even larger.

### Fractional atomic coordinates and isotropic or equivalent isotropic displacement parameters (Å^2^)

**Table:**

	*x*	*y*	*z*	*U*_iso_*/*U*_eq_	Occ. (<1)
S2	0.86759 (10)	0.22270 (15)	1.02672 (6)	0.0941 (4)	
O1	0.6522 (2)	0.3191 (3)	0.93576 (13)	0.0697 (6)	
N1	0.6491 (2)	0.2726 (3)	0.80711 (15)	0.0627 (7)	
N2	0.7564 (2)	0.2228 (3)	0.86200 (15)	0.0628 (6)	
N3	0.8999 (2)	0.2802 (4)	0.79288 (16)	0.0707 (7)	
C1	0.7617 (3)	0.2516 (4)	0.94048 (19)	0.0672 (8)	
C2	0.5908 (3)	0.3288 (4)	0.85393 (19)	0.0620 (8)	
C3	0.4743 (3)	0.4013 (4)	0.8291 (2)	0.0651 (8)	
C4	0.413 (2)	0.451 (3)	0.8829 (15)	0.093 (8)	0.651 (5)
H4A	0.4370	0.4340	0.9396	0.111*	0.651 (5)
C5	0.2989 (18)	0.542 (3)	0.8296 (11)	0.083 (5)	0.651 (5)
H5A	0.2436	0.5951	0.8503	0.100*	0.651 (5)
C6	0.2904 (15)	0.533 (2)	0.7508 (13)	0.092 (5)	0.651 (5)
H6A	0.2254	0.5768	0.7096	0.110*	0.651 (5)
S1	0.4025 (5)	0.4400 (7)	0.7304 (4)	0.0799 (9)	0.651 (5)
S1A	0.4022 (12)	0.4715 (16)	0.8908 (8)	0.0785 (19)	0.349 (5)
C6A	0.293 (3)	0.522 (6)	0.825 (3)	0.125 (17)	0.349 (5)
H6AA	0.2263	0.5592	0.8375	0.150*	0.349 (5)
C5A	0.292 (3)	0.512 (4)	0.745 (2)	0.112 (13)	0.349 (5)
H5AA	0.2322	0.5508	0.6995	0.135*	0.349 (5)
C4A	0.416 (3)	0.420 (5)	0.745 (2)	0.090 (13)	0.349 (5)
H4AA	0.4405	0.3878	0.7008	0.108*	0.349 (5)
C7	0.8480 (3)	0.1488 (4)	0.83120 (19)	0.0694 (8)	
H7A	0.9088	0.0953	0.8767	0.083*	
H7B	0.8143	0.0545	0.7918	0.083*	
C8	0.9964 (3)	0.3838 (6)	0.8467 (2)	0.0919 (12)	
H8A	0.9945	0.5035	0.8255	0.138*	
H8B	1.0697	0.3280	0.8492	0.138*	
H8C	0.9887	0.3883	0.9010	0.138*	
C9	0.8762 (3)	0.2882 (4)	0.70804 (19)	0.0638 (8)	
C10	0.9562 (3)	0.3627 (4)	0.6735 (2)	0.0806 (10)	
H10A	1.0280	0.4050	0.7073	0.097*	
C11	0.9293 (5)	0.3740 (6)	0.5890 (3)	0.1074 (15)	
H11A	0.9838	0.4249	0.5669	0.129*	
C12	0.8256 (6)	0.3131 (7)	0.5371 (3)	0.1167 (17)	
H12A	0.8090	0.3218	0.4803	0.140*	
C13	0.7459 (4)	0.2383 (5)	0.5709 (3)	0.0997 (14)	
H13A	0.6744	0.1965	0.5363	0.120*	
C14	0.7697 (3)	0.2240 (4)	0.6550 (2)	0.0766 (9)	
H14A	0.7150	0.1717	0.6764	0.092*	

### Atomic displacement parameters (Å^2^)

**Table:**

	*U*^11^	*U*^22^	*U*^33^	*U*^12^	*U*^13^	*U*^23^
S2	0.0998 (8)	0.1138 (8)	0.0625 (6)	0.0053 (5)	0.0162 (5)	−0.0036 (5)
O1	0.0771 (16)	0.0794 (14)	0.0594 (13)	−0.0076 (10)	0.0311 (12)	−0.0051 (9)
N1	0.0636 (17)	0.0670 (14)	0.0627 (15)	−0.0058 (12)	0.0270 (14)	−0.0015 (11)
N2	0.0631 (18)	0.0750 (15)	0.0545 (14)	−0.0025 (12)	0.0245 (14)	−0.0021 (11)
N3	0.0626 (17)	0.0918 (18)	0.0619 (16)	−0.0176 (12)	0.0255 (14)	−0.0119 (13)
C1	0.071 (2)	0.0719 (19)	0.0627 (19)	−0.0063 (14)	0.0268 (18)	−0.0024 (13)
C2	0.068 (2)	0.0644 (18)	0.0607 (18)	−0.0118 (14)	0.0297 (17)	−0.0054 (13)
C3	0.063 (2)	0.0739 (19)	0.065 (2)	−0.0117 (14)	0.030 (2)	−0.0079 (15)
C4	0.076 (11)	0.096 (9)	0.093 (13)	−0.001 (6)	0.010 (8)	−0.011 (6)
C5	0.092 (12)	0.080 (6)	0.099 (9)	−0.013 (5)	0.059 (9)	−0.012 (5)
C6	0.067 (9)	0.082 (6)	0.105 (11)	0.002 (5)	−0.002 (7)	0.002 (6)
S1	0.071 (2)	0.1025 (17)	0.0634 (11)	−0.0140 (14)	0.0171 (11)	−0.0039 (14)
S1A	0.082 (4)	0.087 (3)	0.082 (3)	−0.006 (3)	0.049 (3)	−0.006 (3)
C6A	0.044 (17)	0.13 (3)	0.20 (3)	0.002 (14)	0.03 (2)	−0.03 (2)
C5A	0.077 (18)	0.18 (3)	0.09 (2)	−0.040 (14)	0.050 (15)	0.024 (17)
C4A	0.044 (12)	0.130 (18)	0.11 (3)	0.011 (9)	0.041 (15)	0.031 (12)
C7	0.070 (2)	0.0747 (19)	0.067 (2)	0.0020 (15)	0.0258 (17)	0.0004 (15)
C8	0.077 (3)	0.115 (3)	0.080 (2)	−0.026 (2)	0.020 (2)	−0.016 (2)
C9	0.065 (2)	0.0672 (17)	0.0646 (19)	0.0082 (14)	0.0283 (17)	−0.0042 (13)
C10	0.088 (3)	0.078 (2)	0.090 (3)	0.0077 (17)	0.048 (2)	0.0057 (17)
C11	0.147 (4)	0.096 (3)	0.109 (4)	0.034 (3)	0.082 (3)	0.026 (3)
C12	0.175 (5)	0.110 (4)	0.072 (3)	0.050 (3)	0.049 (3)	0.013 (2)
C13	0.117 (4)	0.099 (3)	0.072 (2)	0.027 (2)	0.014 (3)	−0.010 (2)
C14	0.080 (2)	0.080 (2)	0.070 (2)	0.0079 (17)	0.024 (2)	−0.0098 (16)

### Geometric parameters (Å, º)

**Table:**

S2—C1	1.632 (3)	C6A—C5A	1.35 (6)
O1—C2	1.364 (3)	C6A—H6AA	0.9300
O1—C1	1.382 (4)	C5A—C4A	1.63 (5)
N1—C2	1.283 (4)	C5A—H5AA	0.9300
N1—N2	1.385 (3)	C4A—H4AA	0.9300
N2—C1	1.339 (4)	C7—H7A	0.9700
N2—C7	1.464 (4)	C7—H7B	0.9700
N3—C9	1.388 (4)	C8—H8A	0.9600
N3—C7	1.423 (4)	C8—H8B	0.9600
N3—C8	1.455 (4)	C8—H8C	0.9600
C2—C3	1.432 (4)	C9—C10	1.386 (4)
C3—C4	1.388 (19)	C9—C14	1.400 (5)
C3—C4A	1.39 (3)	C10—C11	1.379 (5)
C3—S1A	1.641 (12)	C10—H10A	0.9300
C3—S1	1.663 (6)	C11—C12	1.360 (6)
C4—C5	1.55 (3)	C11—H11A	0.9300
C4—H4A	0.9300	C12—C13	1.377 (6)
C5—C6	1.32 (3)	C12—H12A	0.9300
C5—H5A	0.9300	C13—C14	1.378 (5)
C6—S1	1.641 (19)	C13—H13A	0.9300
C6—H6A	0.9300	C14—H14A	0.9300
S1A—C6A	1.49 (5)		
			
C2—O1—C1	106.2 (2)	C6A—C5A—C4A	107 (3)
C2—N1—N2	103.6 (2)	C6A—C5A—H5AA	126.3
C1—N2—N1	112.3 (3)	C4A—C5A—H5AA	126.3
C1—N2—C7	127.7 (3)	C3—C4A—C5A	102 (2)
N1—N2—C7	119.9 (2)	C3—C4A—H4AA	128.9
C9—N3—C7	122.2 (3)	C5A—C4A—H4AA	128.9
C9—N3—C8	120.1 (3)	N3—C7—N2	112.9 (3)
C7—N3—C8	116.7 (3)	N3—C7—H7A	109.0
N2—C1—O1	104.5 (3)	N2—C7—H7A	109.0
N2—C1—S2	131.6 (3)	N3—C7—H7B	109.0
O1—C1—S2	123.9 (2)	N2—C7—H7B	109.0
N1—C2—O1	113.4 (3)	H7A—C7—H7B	107.8
N1—C2—C3	127.3 (3)	N3—C8—H8A	109.5
O1—C2—C3	119.3 (3)	N3—C8—H8B	109.5
C4—C3—C4A	116.9 (19)	H8A—C8—H8B	109.5
C4—C3—C2	124.6 (12)	N3—C8—H8C	109.5
C4A—C3—C2	118.5 (15)	H8A—C8—H8C	109.5
C4A—C3—S1A	115.5 (15)	H8B—C8—H8C	109.5
C2—C3—S1A	126.0 (6)	C10—C9—N3	121.1 (3)
C4—C3—S1	114.3 (12)	C10—C9—C14	118.1 (3)
C2—C3—S1	121.0 (3)	N3—C9—C14	120.7 (3)
S1A—C3—S1	112.7 (6)	C11—C10—C9	120.1 (4)
C3—C4—C5	106.2 (16)	C11—C10—H10A	119.9
C3—C4—H4A	126.9	C9—C10—H10A	119.9
C5—C4—H4A	126.9	C12—C11—C10	122.1 (4)
C6—C5—C4	110.7 (15)	C12—C11—H11A	119.0
C6—C5—H5A	124.7	C10—C11—H11A	119.0
C4—C5—H5A	124.7	C11—C12—C13	118.2 (4)
C5—C6—S1	115.3 (13)	C11—C12—H12A	120.9
C5—C6—H6A	122.4	C13—C12—H12A	120.9
S1—C6—H6A	122.4	C12—C13—C14	121.4 (5)
C6—S1—C3	93.3 (8)	C12—C13—H13A	119.3
C6A—S1A—C3	96.2 (18)	C14—C13—H13A	119.3
C5A—C6A—S1A	118 (3)	C13—C14—C9	120.1 (4)
C5A—C6A—H6AA	120.8	C13—C14—H14A	120.0
S1A—C6A—H6AA	120.8	C9—C14—H14A	120.0
			
C2—N1—N2—C1	−0.7 (3)	C2—C3—S1—C6	−175.1 (6)
C2—N1—N2—C7	179.4 (2)	S1A—C3—S1—C6	−0.3 (8)
N1—N2—C1—O1	1.3 (3)	C4A—C3—S1A—C6A	3 (3)
C7—N2—C1—O1	−178.9 (3)	C2—C3—S1A—C6A	−179.0 (19)
N1—N2—C1—S2	−177.7 (2)	S1—C3—S1A—C6A	6 (2)
C7—N2—C1—S2	2.1 (5)	C3—S1A—C6A—C5A	−6 (4)
C2—O1—C1—N2	−1.3 (3)	S1A—C6A—C5A—C4A	7 (5)
C2—O1—C1—S2	177.8 (2)	C4—C3—C4A—C5A	4 (3)
N2—N1—C2—O1	−0.2 (3)	C2—C3—C4A—C5A	−177.8 (14)
N2—N1—C2—C3	178.2 (3)	S1A—C3—C4A—C5A	1 (3)
C1—O1—C2—N1	1.0 (3)	S1—C3—C4A—C5A	−52 (19)
C1—O1—C2—C3	−177.6 (3)	C6A—C5A—C4A—C3	−4 (4)
N1—C2—C3—C4	176.5 (11)	C9—N3—C7—N2	−106.3 (3)
O1—C2—C3—C4	−5.2 (11)	C8—N3—C7—N2	85.5 (4)
N1—C2—C3—C4A	−2.0 (17)	C1—N2—C7—N3	−107.6 (3)
O1—C2—C3—C4A	176.4 (17)	N1—N2—C7—N3	72.2 (3)
N1—C2—C3—S1A	179.8 (5)	C7—N3—C9—C10	−154.3 (3)
O1—C2—C3—S1A	−1.9 (6)	C8—N3—C9—C10	13.5 (4)
N1—C2—C3—S1	−6.1 (5)	C7—N3—C9—C14	26.8 (4)
O1—C2—C3—S1	172.2 (3)	C8—N3—C9—C14	−165.3 (3)
C4A—C3—C4—C5	−8 (2)	N3—C9—C10—C11	−178.1 (3)
C2—C3—C4—C5	173.5 (10)	C14—C9—C10—C11	0.8 (5)
S1A—C3—C4—C5	55 (29)	C9—C10—C11—C12	−0.3 (6)
S1—C3—C4—C5	−4.1 (17)	C10—C11—C12—C13	0.1 (6)
C3—C4—C5—C6	4 (2)	C11—C12—C13—C14	−0.3 (6)
C4—C5—C6—S1	−3 (2)	C12—C13—C14—C9	0.8 (5)
C5—C6—S1—C3	0.2 (15)	C10—C9—C14—C13	−1.0 (5)
C4—C3—S1—C6	2.6 (12)	N3—C9—C14—C13	177.9 (3)
C4A—C3—S1—C6	129 (20)		
